# Learning from humans to build social cognition among robots

**DOI:** 10.3389/frobt.2023.1030416

**Published:** 2023-02-06

**Authors:** Nicolas Coucke, Mary Katherine Heinrich, Axel Cleeremans, Marco Dorigo

**Affiliations:** ^1^ IRIDIA, Université Libre de Bruxelles, Brussels, Belgium; ^2^ Consciousness, Cognition and Computation Group, Center for Research in Cognition and Neurosciences, Université Libre de Bruxelles, Brussels, Belgium

**Keywords:** artificial social cognition, embodied cognition, self-organization, robot swarms, multi-robot systems, artificial intelligence, artificial general intelligence, social robots

## Abstract

Self-organized groups of robots have generally coordinated their behaviors using quite simple social interactions. Although simple interactions are sufficient for some group behaviors, future research needs to investigate more elaborate forms of coordination, such as social cognition, to progress towards real deployments. In this perspective, we define social cognition among robots as the combination of social inference, social learning, social influence, and knowledge transfer, and propose that these abilities can be established in robots by building underlying mechanisms based on behaviors observed in humans. We review key social processes observed in humans that could inspire valuable capabilities in robots and propose that relevant insights from human social cognition can be obtained by studying human-controlled avatars in virtual environments that have the correct balance of embodiment and constraints. Such environments need to allow participants to engage in embodied social behaviors, for instance through situatedness and bodily involvement, but, at the same time, need to artificially constrain humans to the operational conditions of robots, for instance in terms of perception and communication. We illustrate our proposed experimental method with example setups in a multi-user virtual environment.

## Introduction

AI research has greatly advanced, but when interaction with other agents is required, existing algorithms easily break down (Bard et al., 2020). Social interaction and social embodiment are still underexplored in artificial general intelligence (Bolotta and Dumas, 2022) and in groups of intelligent robots. While there is some robotics research on social cognition, it focuses on human-robot interaction (Henschel et al., 2020), e.g., how a robot interprets the intentions of a human, not on interactions *among* robots. It is important to note that what looks like social cognition is not necessarily social cognition. For instance, agents or robot controllers made by reinforcement learning might behave in ways that look socially cognizant in some situations, but this might only be appearance—i.e., the underlying behavioral phenomena are not there—so the illusion will break down when exposed to more situations.

Robots can coordinate with each other by using, e.g., centralized control or self-organization. In multi-robot systems that are not self-organized, robots are directed to follow a centrally coordinated plan using explicit commands or global references. In this paper, we are interested exclusively in robot groups that include aspects of self-organization, because social cognition depends on some degree of individual autonomy. If a robot is essentially a remote-controlled sensor or actuator, it does not engage in social cognition.

In existing research on self-organized robot groups, the individuals are usually quite simple and often rely on indiscriminate, naïve interactions. Indeed, swarm robotics research has shown that no advanced cognition or elaborate social negotiation is needed to self-organize certain group behaviors (e.g., Nouyan et al., 2009; Rubenstein et al., 2014; Valentini et al., 2016). However, it has been argued that there are still significant gaps for robot swarms to be deployment-ready, and that the future of swarm robotics research should concentrate on more elaborate forms of self-organized coordination (Dorigo et al., 2020; 2021), such as self-organized hierarchy (Mathews et al., 2017; Zhu et al., 2020) or behavioral heterogeneity (Kengyel et al., 2015).

In this perspective, we argue that another important direction for future study should be social cognition. Robot groups successfully equipped with social cognition could engage in elaborate coordination without sending each other large amounts of data. Some aspects of robot behavior could be mutually predictable, for instance by robots maintaining good internal models of each other. Socially cognitive robots could have improved group performance, e.g., by not destructively interfering with each other (which requires time and effort to resolve) and not accidentally disrupting each other’s sub-goals while attempting to reach a common goal.

In cognitive robotics, research on individual robots such as humanoids is very advanced (Cangelosi and Asada, 2022), even on each of the six key attributes of artificial cognitive systems (Vernon, 2014): action, perception, autonomy, adaptation, learning, and anticipation. Comparatively, cognition in swarm robotics is still in its beginning stages. While cognitive robot swarms can be autonomously capable of collective action, perception, and in some cases adaptation (Heinrich et al., 2022), we do not yet know how to make robot swarms that can autonomously learn and anticipate as a collective, in such a way that the group behavior is greater than the sum of its parts. We propose that studying social cognition could help us advance the autonomous collective capabilities of groups of robots.

## Socially cognitive robots: Our perspective

Our perspective is summarized as follows: social cognition among robots can be built by developing artificial social reasoning capabilities based on behaviors observed in humans.

Frith (2008) has defined social cognition in humans as “the various psychological processes that enable individuals to take advantage of being part of a social group” and Frith and Frith (2012) have further specified that a substantial portion of these psychological processes are for learning about and making predictions about other members of the social group. The mechanisms of social cognition in humans include social signalling, social referencing, mentalizing (i.e., tracking of others’ mental states, intended actions, objectives, and opinions), observational learning (e.g., social reward learning, mirroring), deliberate knowledge transfer (e.g., teaching), and sharing of experiences through reflective discussion (Frith, 2008; Frith and Frith, 2012). Crucially, social cognition is also defined as “not reducible to the workings of individual cognitive mechanisms” (De Jaegher et al., 2010).

Although some social abilities such as simple social interaction are well-developed among robots, most of the abilities contained in Frith (2008)’s definition of social cognition are lacking, and could provide significant performance benefits. For instance, the transfer of information between robots is well understood, but much less so the transfer of knowledge, especially implicitly:

We define social cognition among robots as the following set of abilities:

1. **Social inference**—inferring the opinions, intended next actions, and overall goals of other robots in the same social group, using interpretation of social signals;
2. **Social learning**—learning information about which actions to adopt or avoid based on observations of each other’s behaviors and social signalling;
3. **Social influence**—deliberately influencing each other’s (socially inferred) internal states using social signaling; and
4. **Knowledge transfer**—transferring high-level knowledge using social interaction, e.g., using implicit demonstration or explicit instruction.

Currently, robots are well-equipped with some of the requirements for these abilities, such as simple social interactions, but lack other crucial requirements such as explicit social reasoning. Although research has shown that no social cognition is needed for simple group behaviors in robots, it is an open challenge how to accomplish more advanced behaviors in a fully self-organized way. Some of the significant unresolved technical challenges for advanced self-organization among robots, which we believe social cognitive abilities could contribute to, are the following:

• autonomously anticipating which actions should be taken in an environment filled with other autonomous robots,
• collectively defining an explicit goal that was not pre-programmed and collectively directing the robot group towards it,
• making online inferences about other robots’ current states and future behaviors, and adapting their coordination strategies accordingly, even while moving at high speed in dynamic unknown environments, and
• designing self-organization among robots such that the resulting group behaviors, although not completely predictable, are safe and trustable.

We propose that socially cognitive robots can in part be developed by learning from the social cognition processes of humans in certain experimental conditions. In order to have the potential to transfer observed behaviors and capabilities from humans to robots, we believe experiments with human subjects must be conducted in a platform that allows experimental setups to be: on one hand, realistic enough to study **embodied** human behavior, but on the other hand, **constrained** and simplified enough to approximate the operational conditions of robots.

## State of the art

### Artificial social learning and artificial mentalizing

Many examples of artificial learning exist that seem relevant to the mechanisms of social cognition. However, key social aspects are not present in these existing methods: for instance, reward learning has been demonstrated in robots (e.g., Daniel et al., 2015) but learning of social rewards among robots has not been studied. Likewise, robots learning by interacting with and observing other robots has been demonstrated (e.g., Murata et al., 2015), but not for the learning of socially relevant information nor to build behaviors among robots that are irreducible to the knowledge held by robots individually.

Currently, the most advanced research towards artificial social cognition can be seen in multi-agent reinforcement learning. In basic approaches, each agent would use reinforcement learning individually, treating other agents as part of the environment. In more elaborate existing approaches, agents are trained to model each other and several types of artificial mentalizing have been demonstrated (Albrecht and Stone, 2018). For example, in the Deep Reinforcement Opponent Network (DRON), one agent learns the representation of the opponent’s policy (He et al., 2016). In another example, an agent uses itself as the basis to predict another agent’s actions (Raileanu et al., 2018). One approach using a “Theory of Mind” network has even produced agents that can explicitly report inferred mental states of other agents and pass the classic “false belief test” for understanding the mental states of others (Rabinowitz et al., 2018). Current efforts in multi-agent learning use cooperative games such as Hanabi as benchmarks, which involves inferring the mental states of others and using that information to collaborate (Bard et al., 2020). For the development of artificial social cognition, the next step for this line of research would be to situate the mentalizing behaviors within the full set of social cognition mechanisms, including social influence and social reward learning (*cf.* Olsson et al., 2020).

### Social cognition transfer between humans and robots

Robots have been used as experimental tools for the study of embodied social cognition. For instance, a variety of devices have been used to automatically provide synthetic social stimuli to animals in a naturalistic way (Frohnwieser et al., 2016). Similarly, the effect of humanoid robots on human social cognition has been broadly studied (Wykowska et al., 2016). Social robots in the context of human-robot interaction have also been investigated (e.g., Dautenhahn, 2007). However, to the best of our knowledge, no studies have looked at expanding these robot use cases into embodied artificial social cognition among robots, and no work apart from our own has proposed using experiments with humans to contribute to building social cognition among robots.

## Directions for future research

Advanced group capabilities seen in humans can inspire similar capabilities in robots. For example, the human capabilities of selecting and following leaders (Van Vugt, 2006) and re-organizing communication networks around individuals with better information (Almaatouq et al., 2020) have recently inspired the development of self-organized hierarchies for robots, for instance using physical (Mathews et al., 2017) or wireless connections (Zhu et al., 2020). In the following sections, we identify cognitive processes used by humans in social situations that would be valuable for robot groups, and propose them as future research directions for building social cognition among robots.

### Social heuristics and action selection

Humans often use cognitive processes known as “heuristics” to select actions in social situations. In humans, heuristics are defined as action selection strategies that usually deviate from economic rationality or Bayesian optimality but which facilitate a rapid action selection when time and knowledge about a situation are limited (Hertwig and Herzog, 2009). The hidden states of other agents cannot be directly observed, so the outcome of a social situation always has a high degree of uncertainty—selecting the optimal action is computationally intractable (Seymour and Dolan, 2008).

In humans, heuristics can involve continuous integration of multiple variables or sources of information, for example when deciding on a walking direction based on the position of other walking individuals (Moussaid et al., 2011). In psychology and neuroscience, action selection is often characterized as the result of an accumulation process, in which evidence that supports a certain decision or action is accumulated over time (Ratcliff and McKoon, 2008). A certain action is taken when the accumulated evidence crosses some threshold. The sources and manner of evidence integration can be determined by social heuristics. For example, evidence accumulation frameworks can characterize how humans use a “follow the majority heuristic” during social decision making (Tump et al., 2020), as well as how humans base their own movements on those of others during embodied competitive interactions (Lokesh et al., 2022).

### Coupling, alignment, and mirroring

Humans often mirror each other’s behaviors and can participate in a “coupling” behavior through reciprocal interactions. Implicit coupling can occur between physiological states (for example, synchronization of heartbeats and breathing rhythms). Explicit sensorimotor coupling involves mutual prediction of each other’s actions and facilitates coordinated action sequences (Dumas and Fairhurst, 2021). On a higher cognitive level, reciprocal interactions can create alignment between internal cognitive states, which in turn facilitates better mutual prediction of actions (Friston and Frith, 2015).

Humans can also disengage from social interactions and instead mirror (or “simulate”) others’ actions as a type of internalized action (Buzsáki, 2019, p. 131). This capacity is supported by the mirror neuron system, which is active when observing and when executing a movement (Rizzolatti and Craighero, 2004). Internal simulation aids in understanding others’ intentions and in selecting complementary actions (Newman-Norlund et al., 2007).

### Mentalizing and shared representations

Simply mirroring the mental states of others is often not sufficient to infer their opinions, objectives, or intended actions (Saxe, 2005). Therefore, coupling and mirroring are often complemented in humans by higher-level cognition about others’ beliefs, desires, and intentions, taking into account factors such as context and memory (Sebanz et al., 2006). This requires mentalizing, a process of inference about others’ changing mental states, beyond simple mirroring (Frith and Frith, 2012).

For example, mentalizing based on observations of others’ gazes facilitates taking others’ perspectives into account and tracking their beliefs about a shared environment or world (Frith and Frith, 2012). By observing others’ movements, humans can also infer the confidence that others have in their beliefs (Patel et al., 2012) and the intentions that underlie their actions (Baker et al., 2009). Crucially, humans also mentalize based on third-party observations of others’ interactions, and then estimate the social relationships between them (Ullman et al., 2009).

Tracking others’ goals and beliefs helps humans to distinguish which subset of their action representations are shared with others. Shared representations aid in predicting and interpreting the actions of others in the context of a joint goal, and in selecting complementary actions. For instance, by tracking others’ beliefs, an individual can recognize when communication or signalling is needed to facilitate smooth coordination (Pezzulo and Dindo, 2011).

### Outcome monitoring

Humans monitor behaviors and detect errors when taking actions directed towards a certain goal (Botvinick et al., 2001). If an individual recognizes another making what might be an error, in pursuit of a shared goal, the individual needs to then distinguish whether it was indeed an error, or whether their goals are misaligned.

Humans also monitor whether actions have their intended outcomes, as well as whether a certain action and certain outcome actually have a causal link. This results in a greater or lesser sense of agency over a certain action or outcome (Haggard and Chambon, 2012), which in turn impacts how an individual acts in social situations. Agency can be modulated in a variety of ways: joint agency when acting together with others, vicarious agency when influencing the actions of others, or violated agency when actions are interfered with by others (Silver et al., 2020). The modulated sense of agency in humans helps shape an individual’s monitoring of links between actions, errors, and outcomes.

## From humans to robots: An experimental method

Robots are embodied agents with specific morphologies and specific perception and action capabilities that differ from (and are often far more limited than) those of humans. To gain insights from human social cognition that are relevant to robots, human subjects would need to be studied in an experimental platform that: 1) allows them to engage in embodied social behaviors, but also 2) allows enough constraints to artificially expose humans to the operational conditions of robots. We propose that behavioral experiments conducted with humans controlling avatars in virtual environments can achieve this trade-off.

### Balancing embodiment and constraints in virtual environments

Existing experiments on human social cognition have mostly been conducted in highly controlled single-person paradigms which lack embodiment. We identify the following five aspects of embodiment that we propose human-controlled avatars in new virtual environments will need, for the study of embodied human social cognition.

1. **Situatedness**: An agent takes actions while being part of a situation, rather than by observing the situation from the outside (Wilson, 2002).
2. **Sensory and action shaping**: By taking actions (e.g., moving their bodies) in the environment, agents can actively change the flow of their sensory inputs as well as the potential effects of their actions (Gordon et al., 2021).
3. **Bodily involvement**: The bodily state and/or morphology of the agent—as well as the agent’s bodily relation to the bodies of other agents—can be involved in cognition (Wilson, 2002).
4. **Interaction cascades**: Agents can engage with each other in such a way that actions by one can influence reciprocal actions by another, resulting in cascades of interactions and behaviors (Dale et al., 2013).
5. **High bandwidth**: There can be high bandwidth of implicit or explicit information exchange between agents (Schilbach et al., 2013).

Complementarily, we identify the following constraints that will also need to be possible in the virtual environment.

1. **Body and action**: Human-controlled avatars can be equipped with morphology features and action capabilities that are similar to those of relevant robots.
2. **Perception**: When controlling an avatar, a human subject can be limited to sensory inputs similar to those of relevant robots (e.g., restricted visual information).
3. **Communication**: Human-controlled avatars can be limited to communication and signalling capabilities that are similar to those available to relevant robots.
4. **Hidden states**: Human subjects can be required to explicitly report information about hidden states (e.g., their current opinion or confidence level) that is not directly observable from their behavior but would be available to an experimenter if using relevant robots.

Unconstrained real-world social situations would fulfill all listed requirements for embodiment, but would lack control and interpretability. Virtual environments enable certain aspects of embodiment while at the same time ensuring a degree of control of the situation for the experimenter.

### Example: Using the virtual environment HuGoS

To the best of our knowledge, no off-the-shelf virtual environment was available to meet these requirements, so we built a tool in Unity3D called “HuGoS: Humans Go Swarming” (Coucke et al., 2020; 2021) that we could use to study human behavior in embodied scenarios similar to those in which robots operate. To illustrate the features that we propose for a virtual environment for studying transferable social cognition, we describe two example experimental setups in HuGoS.

#### Collective decision making

**FIGURE 1:**
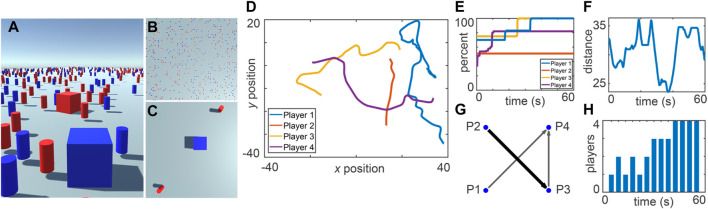
Collective decision making. Participants control cubic avatars while having either a broad **(A)** or limited **(C)** view of the full environment **(B)**. A wide variety of variables can be measured during the experiment, such as the participants’ trajectories **(D)**, the percentage of the environment they have explored **(E)**, the average distance between participants **(F)**, the participant-participant viewing network **(G)**, and the number of avatars choosing the correct color **(H)**. Figure reprinted by permission from Springer Nature Customer Service Centre GmbH: Springer eBook, Coucke et al. (2020), © Springer Nature 2020.

Collective decision making has been widely studied in swarm robotics (Valentini et al., 2017), but many gaps still remain (Khaluf et al., 2019). Collective decisions have also been extensively studied in humans (Kameda et al., 2022), but not typically in embodied scenarios that would be relevant to robots, in which, e.g., exploration and signalling can take place simultaneously. In our example implementation in Coucke et al. (2020), each of four participants controls the movements of a cubic avatar in an environment scattered with red and blue cylindrical landmarks (see Figure 1). The task is to explore the environment while making observations through the avatar’s (broad or limited) field of view and simultaneously deciding whether there are more red or blue landmarks present in the environment. The participants must come to a consensus in order to complete the task and are only permitted to communicate with each other indirectly: they vote by changing their avatar color and they observe the avatar colors of the other participants while making their decisions (see Figures 1A–C). During an experiment, all perceptual information available to each participant, along with their actions, are recorded at a sampling rate of 10 Hz (Figures 1D–H).

In this experiment setup, participants came to a consensus about the predominant color in the environment through a combination of environmental and social information. In the example trial shown in Figure 1, at 45 s, all four participants had adopted the correct opinion (Figure 1H) after individually and broadly exploring the environment and then reducing their average relative distances to increase their access to social information (Figure 1F) and finally come to a consensus. When a consensus was reached, not all participants had personally observed all parts of the environment (Figure 1E), implying that social information was effectively used. Further, all participants had a strong directional line-of-sight connection with at least one other participant (Figure 1G), but the most looked-at participant (P4) had not personally observed the whole environment (Figure 1E), implying that the consensus on the correct opinion was indeed arrived at by a self-organized and collective process. For more information on this and similar experiments, please refer to Coucke et al. (2020). By setting up more advanced experiments in this direction, data could be collected to, for example, identify social heuristics that can inspire new protocols in future robot swarms.

#### Collective construction

**FIGURE 2:**
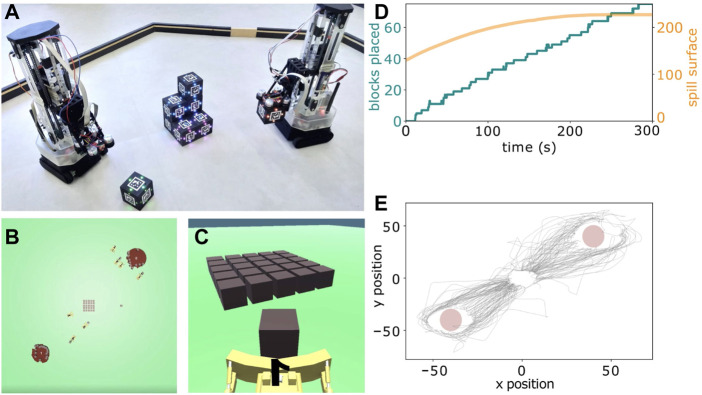
Collective construction. **(A)** Two physical robots that perform collective construction using stigmergic blocks (Allwright et al., 2019). Figure **(A)** reprinted from Allwright et al. (2019) under license CC BY-NC-ND 4.0. **(B, C)** “Lava spill task” in which participants use indirect communication to collectively construct a barrier to contain expanding spills. **(D)** The spill size stagnates after around 200 s, when participants successfully enclosed it with construction blocks. **(E)** Data such as the avatar trajectories can be used to analyze how participants coordinate the placement of blocks. Figures **(B–D)** adapted from Coucke et al. (2021) under license CC BY 4.0.

Existing swarm robotics approaches to construction often use stigmergy (i.e., indirect communication through modification of the environment) to coordinate (Petersen et al., 2019), but the structures built strictly by stigmergy are relatively simple. Future robot swarms should be able to build complex structures in dynamically changing environments (Dorigo et al., 2020). In our example “lava spill task” scenario in Coucke et al. (2021), human social behaviors in collective construction scenarios can be observed. In this task (see Figure 2), participants are instructed to collectively construct a barrier to contain an expanding spill, but are not instructed how to coordinate. Each participant controls the movement of an avatar that can push construction blocks. The environment includes two different spills (i.e., expanding circles) and a supply of construction blocks placed in between them. During an experiment, a group of eight participants needs to assess the environment and coordinate their actions using indirect communication (i.e., observation of peers) to barricade both of the expanding spills within 300 s.

The avatar trajectories in Figure 2E show that participants coordinated to distribute their work between the two spills and place blocks around the full circumferences of both spills. Figure 2D shows that participants continued to place more blocks at a roughly constant rate throughout the experiment, implying that no bottleneck arose in their self-organized coordination. The figure also shows that the expansion of both spills had successfully been stopped at around 200 s. For more information on this and similar experiments, please refer to Coucke et al. (2021). Using more advanced setups in this direction, the gathered behavioral data could provide insights into how self-organized coordination and group actions unfold over time and adapt to the environment. In order to get detailed information about participant strategies, experiments in this virtual environment can be temporarily interrupted at certain times to ask participants about, e.g., their explicit judgements about the beliefs of other participants, their sense of (joint) agency, or their feelings of alignment with others.

## Discussion

Some features of human social groups, such as collective intentions, reflective discussion, or shared biases, might at first seem not particularly relevant for robots. However, there are many autonomous group behaviors that have not yet been demonstrated in self-organized robots. For instance, it is not yet understood how to have robots autonomously identify when they should make a collective decision (Khaluf et al., 2019). These fundamentals of group-level autonomy, which social animals such as humans exhibit effortlessly and consistently, might possibly be based on, or even depend on, such unexpected features as shared biases. Our perspective is that research that investigates the transfer of such social traits from humans to robots can help us to identify and understand the basic elements needed to build artificial social cognition.

Artificial restrictions in embodied experiments are unlikely to reveal how humans would behave in natural conditions, but there is existing evidence that such restrictions indeed have the potential to reveal aspects of embodied human social behavior that would be transferable to robots. For example, when realistic social cues such as gaze and facial expressions are inhibited, humans have been shown to focus on other communication channels, such as implicit movement-based communication (Roth et al., 2016).

If eventually achieved, the creation of social cognition among robots would open many further research questions. For instance, there are human collective intentions that go beyond the humans that are immediately present (Tomasello et al., 2005)—if robots have advanced social cognition abilities, how should different social groups of robots interact with each other, whether physically or remotely? As another example, intrinsic motivation or curiosity-driven learning could be investigated to motivate agents to explore the complex internal states that make up another agent, perhaps constituting a rudimentary theory of an artificial mind. Or, perhaps robots could be intrinsically motivated to autonomously develop completely new forms of artificial social cognition that do not resemble those already seen in humans or social animals.

## Data Availability

The original contributions presented in the study are included in the article, further inquiries can be directed to the corresponding authors.
